# A Reproducible and Tunable Synthetic Soil Microbial Community Provides New Insights into Microbial Ecology

**DOI:** 10.1128/msystems.00951-22

**Published:** 2022-12-06

**Authors:** Joanna Coker, Kateryna Zhalnina, Clarisse Marotz, Deepan Thiruppathy, Megan Tjuanta, Gavin D’Elia, Rodas Hailu, Talon Mahosky, Meagan Rowan, Trent R. Northen, Karsten Zengler

**Affiliations:** a Department of Pediatrics, University of California, San Diego, La Jolla, California, USA; b Environmental Genomics and Systems Biology Division, Berkeley Lab, Berkeley, California, USA; c Department of Bioengineering, University of California, San Diego, La Jolla, California, USA; d The DOE Joint Genome Institute, Berkeley Lab, Berkeley, California, USA; e Center for Microbiome Innovation, University of California, San Diego, La Jolla, California, USA; Pacific Northwest National Laboratory

**Keywords:** EcoFAB, metagenomics, microbiome, plant-microbe interactions, synthetic communities

## Abstract

Microbial soil communities form commensal relationships with plants to promote the growth of both parties. The optimization of plant-microbe interactions to advance sustainable agriculture is an important field in agricultural research. However, investigation in this field is hindered by a lack of model microbial community systems and efficient approaches for building these communities. Two key challenges in developing standardized model communities are maintaining community diversity over time and storing/resuscitating these communities after cryopreservation, especially considering the different growth rates of organisms. Here, a model synthetic community (SynCom) of 16 soil microorganisms commonly found in the rhizosphere of diverse plant species, isolated from soil surrounding a single switchgrass plant, has been developed and optimized for *in vitro* experiments. The model soil community grows reproducibly between replicates and experiments, with a high community α-diversity being achieved through growth in low-nutrient media and through the adjustment of the starting composition ratios for the growth of individual organisms. The community can additionally be cryopreserved with glycerol, allowing for easy replication and dissemination of this *in vitro* system. Furthermore, the SynCom also grows reproducibly in fabricated ecosystem devices (EcoFABs), demonstrating the application of this community to an existing *in vitro* plant-microbe system. EcoFABs allow reproducible research in model plant systems, offering the precise control of environmental conditions and the easy measurement of plant microbe metrics. Our results demonstrate the generation of a stable and diverse microbial SynCom for the rhizosphere that can be used with EcoFAB devices and can be shared between research groups for maximum reproducibility.

**IMPORTANCE** Microbes associate with plants in distinct soil communities to the benefit of both the soil microbes and the plants. Interactions between plants and these microbes can improve plant growth and health and are therefore a field of study in sustainable agricultural research. In this study, a model community of 16 soil bacteria has been developed to further the reproducible study of plant-soil microbe interactions. The preservation of the microbial community has been optimized for dissemination to other research settings. Overall, this work will advance soil microbe research through the optimization of a robust, reproducible model community.

## INTRODUCTION

The scientific community has developed robust model systems for research in animals, plants, and individual microbes (1, 2). These systems allow for experiments to be repeated and validated across research groups, leading to a body of research that builds on the work of others. However, microbiome research currently lacks widely accepted reproducible model systems, despite the recognition that microbial communities play fundamental roles in biological systems (3–5). Indeed, host organisms and their microbiota are often referred to as one meta-organism, requiring both parts of the system to thrive (6–8). Several groups have worked to develop reproducible microbial systems, such as a microbial chemostat (9), the Lubbock chronic-wound biofilm model (10), *in vitro* gut systems incorporating microbes (11–13), and, most notably, the Altered Schaedler Flora community (14, 15). These systems address important research questions about the interactions between microbes and their host environments. However, they normally do not probe the mechanisms of host-community interactions, particularly in plant-microbe communities under environmental perturbations.

To address specific questions pertaining to the inner workings of microbial communities, researchers must be able to alter the presence and abundance of specific organisms, introduce genetic alterations as necessary, and maintain strict control of growth conditions, such as temperature, humidity, acidity, and light (8, 16–18). At the present time, experiments with synthetic microbial communities are one of the only viable methods by which to design research studies within these constraints (8, 19–21). Although the use of bioengineering tools to introduce specific changes in natural microbial communities shows promise in this area (22–24), these systems still lack the ability to predict the effects of engineering outcomes on the community as a whole (24). Therefore, synthetic microbial communities represent the most suitable approach by which to investigate community dynamics.

Plant-microbiome interactions have been the focus of an increasing number of studies in recent years, especially given their potential to optimize agricultural production through the promotion of plant growth and soil health (5, 8, 25). These studies clearly show that plant microbiome communities are heavily influenced by the location of microbial colonization on the plant (21, 26, 27) and by the host plant genotype (28, 29). Each of these studies, some explicitly and some implicitly, are searching for what has been termed the “minimal microbial community”, that is, the minimal set of organisms required to accurately reproduce natural community functions (16). The number of microbial strains in constructed communities ranged from under 10 (21, 28) to between 20 and 100 (27, 29–32), although some studies starting with a large number of microbes reported that only a small number of organisms consistently colonized plant sites (21, 30). In addition to the loss of starting organisms, *in vitro* microbial communities commonly lose α-diversity over time, compared to their starting communities (33–35). The vast majority of synthetic microbial communities are constructed with equal amounts of each organism, although Bai et al. (27) compared an equal ratio (1:1:1:1) of four represented phyla to an unequal ratio (1:1:1:0.25) and found that the final community compositions were similar. However, it has recently been shown that the starting ratios, even in a simple coculture, can have a significant effect on community growth and composition (36, 37). To what extent equal ratios in the starting inoculum produce the most reproducible and diverse synthetic community is still an open question (37). When generating soil communities, we hypothesized that synthetic community α-diversity could be increased by starting with higher proportions of organisms that decrease in abundance during community growth.

Here, we present the generation of a diverse, reproducible, and tunable synthetic microbial community that is composed of soil bacteria isolates obtained from switchgrass agricultural fields. Using a picoliter liquid printer to allow for the precise control of the initial bacterial inoculum, we tested over 20 community starting composition ratios to generate a synthetic community with maximum robustness and α-diversity. We then used this community to probe the effect of DNA from dead cells on sequencing composition results. To further support the reproducibility of this model community, we additionally determined a method for the cryopreservation of the community, thereby enabling it to be stored as stocks and thawed without requiring reconstruction. The 16-member community can readily be applied to fabricated ecosystem (EcoFAB) devices, which allow for reproducible research in model plant systems and offer the precise control of environmental conditions and the easy measurement of plant microbe metrics (18, 38).

## RESULTS

### Strain selection.

Our overall goal was to generate a stable, reproducible microbial community for use with EcoFAB devices to study plant-microbe interactions in the rhizosphere. To this end, we selected 18 microbial strains, isolated from the rhizosphere and bulk soil surrounding a single switchgrass plant, that span the typical diversity found in the rhizosphere of grasses or food crops (Table S1). These organisms represent novel strains of known genera found in the rhizosphere. Two strains were later eliminated to result in a final 16-member community, described below. Although the rhizosphere microbiome differs between geographic sites, studies have suggested that a group of “core” organisms is present across sites and between various grass species (39, 40). The 16-member synthetic community (SynCom) incorporates the core microorganisms represented in our isolate collection in addition to other microbes previously associated with the rhizosphere (Table S1). Additionally, all selected strains are from different genera so as to facilitate community diversity and promote the ease of strain identification through 16S rRNA gene sequencing in the final community.

10.1128/msystems.00951-22.1 TABLE S1 Strain isolates used in this study and examples of plants associated with them in previous publications. Download Table S1, DOCX file, 0.2 MB. Copyright © 2022 Coker et al. 2022 Coker et al. https://creativecommons.org/licenses/by/4.0/ This content is distributed under the terms of the Creative Commons Attribution 4.0 International license.

### Automated assembly of SynComs produces more precise results than does hand assembly.

**FIG 1:**
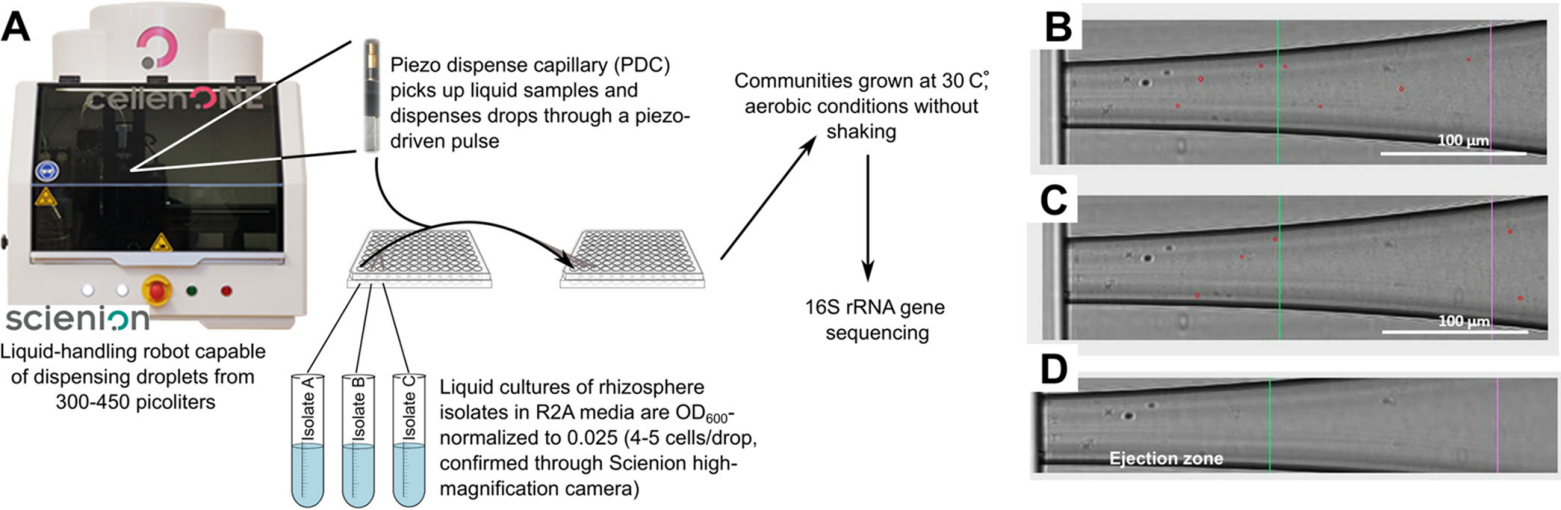
Schematic of synthetic rhizosphere community generation using a liquid-handling machine with a piezo dispense capillary (PDC) device. (A) Isolates were grown for 3 to 4 days in liquid R2A medium, OD_600_-normalized to 0.025, and loaded into individual wells in the probe plate. The PDC drew liquid up from one well of the probe plate and dispensed a programmed number of drops in the desired wells of the target plate. This process was repeated for each isolate to result in a final mixed community. The communities were grown aerobically for the desired amount of time and then analyzed for composition and diversity via 16S rRNA sequencing. (B–D) Individual cells visualized within the nozzle of the PDC prior to dispensing. The machine identifies cells matching a tunable selection criterion (red circles), such as circularity, elongation, and maximum diameter. An initial test prior to generating the communities is shown here, using the machine’s CellenONE module. Droplet generation using various dilutions of the community members was tested to ensure that only 2 to 3 cells were repeatedly detected in the ejection zone of the nozzle (left of the green line) Examples using the organism *Lysobacter* are shown in panels B and C. An OD_600_ dilution to 0.025 of each community member ensured 2 to 3 cells per droplet (droplet volume 390 to 420 pL). (D) Image of the PDC when dispensing sterile PBS. The black dots on the left edge of the ejection zone are background noise on the surface of the nozzle and are not cells.

**FIG 2:**
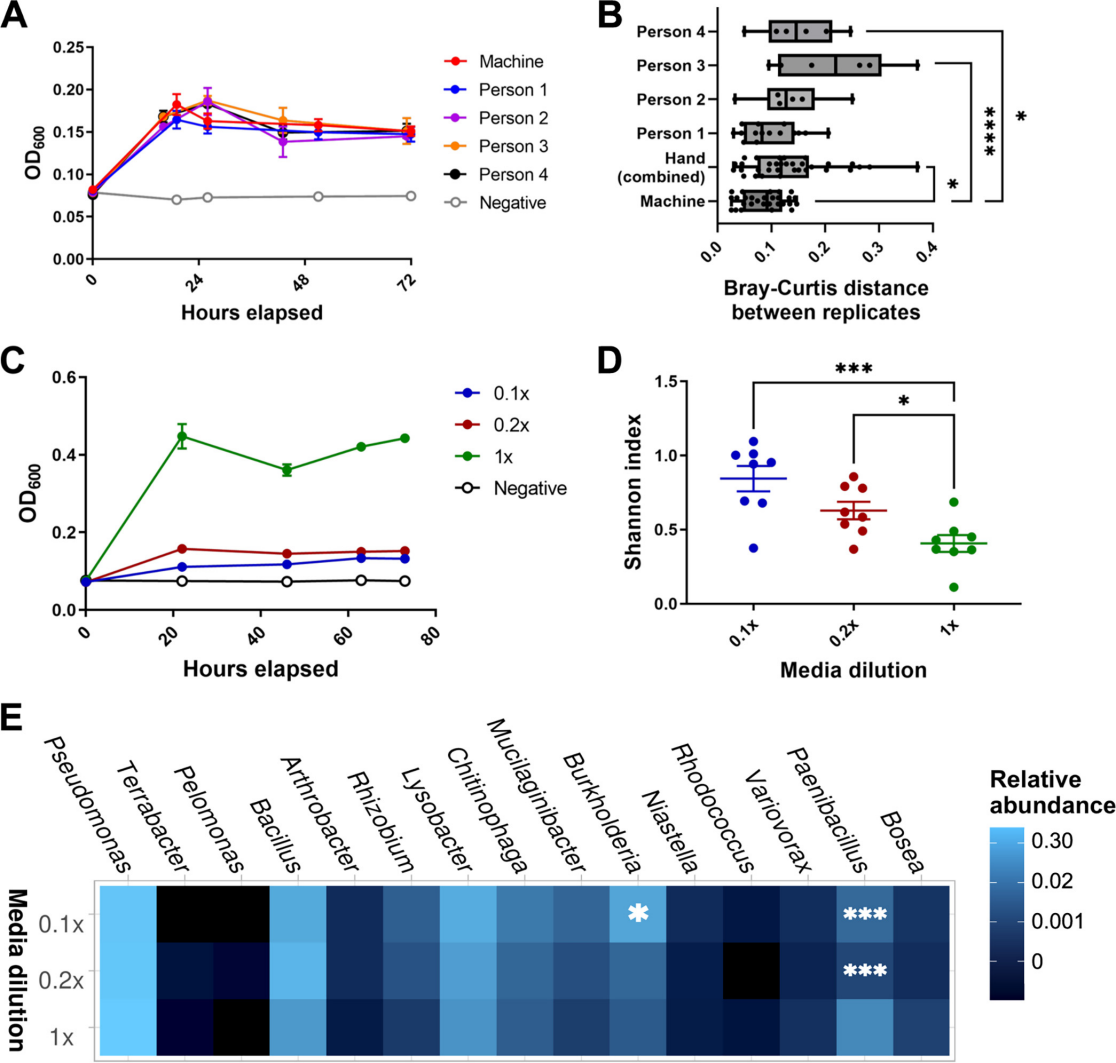
Community diversity with hand-assembly and media dilutions. (A) OD_600_ values of human-assembled (human) and machine-assembled communities (machine) over 3 days (72 h) of growth (*n* = 6 to 8 each). (B) Bray-Curtis distances of 16S rRNA gene amplicon sequencing between replicates of the hand-assembled and machine-assembled communities. The hand-assembled communities are shown combined and broken into the four individuals. (One-way ANOVA with the Benjamini-Hochberg [BH] FDR correction; *, *P* < 0.05; ****, *P* < 0.001) (C) OD_600_ values of the machine-assembled, equally mixed communities in 1×, 0.2×, and 0.1× R2A media over 3 days (*n* = 8 each). (D) Observed OTUs (left) and Shannon diversity index (right) of media dilution communities (Student’s *t* test; *, *P* < 0.05; ***, *P* < 0.005). (E) Heat map of taxonomy relative abundance (genus level) of media dilution communities from 16S sequencing, with the genera differentially abundant from the 1× condition being marked with asterisks (DESeq2 Wald test; fitType = “parametric” with the BH FDR; *, *P*-adj < 0.06; ***, *P*-adj < 0.0001). Taxonomic order was determined via PCoA clustering of the Bray-Curtis distance. Replicates for each condition were merged for the heat map with the Phyloseq command merge_samples (group = “Media_dilution”).

The individual strains were assembled into SynComs using a SCIENION CellenONE liquid-handling machine (Fig. 1). A step-by-step protocol is provided in the Supplemental Material (Text S1). The machine rapidly dispenses individual members of the community using picoliter drops that contain 2 to 3 cells each (Fig. 1B–D), thereby providing increased throughput while minimizing the variability and calibration errors associated with pipetting. We compared the diversity and composition of eight replicates of an automated-assembly SynCom (machine) to 18 replicates of a hand-assembly SynCom (human) after 3 days of growth in 0.1× R2A media. The hand-assembled communities were composed of 4 to 6 replicates each from 4 different lab members. The growth rate and final OD_600_ values were the same between the machine-and hand-assembled communities (Fig. 2A). The Bray-Curtis distance, a β-diversity metric, showed a significantly larger dissimilarity between the SynComs assembled by hand, both for the combined hand-assembled SynComs and for two of the four people, compared to the machine assembly (one-way analysis of variance [ANOVA] with the Benjamini-Hochberg [BH] false discovery rate [FDR] correction, *, *P* < 0.05; ****, *P* < 0.001) (Fig. 2B). Similarly, α-diversity showed a greater spread in the hand-assembled SynComs than in the machine-assembled SynComs for two of the four people (Fig. S1C). These results indicate that SynCom assembly with the automated printer will, on average, result in less variability than will SynCom assembly by different people.

10.1128/msystems.00951-22.4 FIG S1 Comparison of boiling and conventional kit DNA extraction for the rhizosphere isolates (A and B). Isolates were mixed in equal amounts, as determined by the OD normalization. DNA was extracted either by heating to 95°C for 10 minutes in a PCR machine (boiling) or by using a conventional extraction kit (Qiagen PowerSoil Pro) (extraction). (*n* = 3 per condition) (A) Taxonomy of samples through 16S sequencing. (B) Comparison of a logarithmic transformation of the mean relative abundance values in the boiling and extraction samples. Pearson’s correlation coefficient is shown on the plot. Community diversity between assembly methods and media dilutions (C-D). (C) The Shannon diversity index of the machine-assembled and human-assembled communities from 4 different people (*n* = 4 to 8 each). (D) Pielou’s evenness equally mixed community grown in 1×, 0.2×, and 0.1× R2A media for 3 days (*n* = 8 each) (Student’s *t* test; *, *P* < 0.05; **, *P* < 0.01). Download FIG S1, EPS file, 0.6 MB. Copyright © 2022 Coker et al. 2022 Coker et al. https://creativecommons.org/licenses/by/4.0/ This content is distributed under the terms of the Creative Commons Attribution 4.0 International license.

### α-diversity of the SynCom is enhanced through low-nutrient conditions.

Next, we sought to test whether nutrient availability affected the growth of individual strains within the community. We compared the growth and diversity of an equally mixed community of all 18 strains between 1×, 0.2×, and 0.1× R2A media (*n* = 8 for each condition) after 3 days of growth. As expected, the total community growth was highest in the 1× medium, followed by the 0.2× and 0.1× media (Fig. 2C). However, the Shannon diversity index and Pielou’s evenness, two α-diversity metrics, were lowest in the 1× medium and increased as the medium dilution increased (one-way ANOVA with the BH FDR correction; *, *P* < 0.05; ***, *P* < 0.005) (Fig. 2D; Fig. S1D). A taxonomy analysis of the 16S sequencing data revealed that the Pseudomonas strain commonly grew to a high final proportion of the final community, regardless of the medium dilution (Wald test with the BH FDR correction, *, *P* < 0.06; ***, *P* < 0.0001) (Fig. 2E). However, the 0.1× communities displayed higher relative abundances of other, less-dominant strains, such as *Burkholderia*, *Chitinophaga*, and *Mucilaginibacter*, although only the increase in *Burkholderia* was determined to be statistically significant. The individual growth curve of each organism can be found in Fig. S2.

10.1128/msystems.00951-22.5 FIG S2 Growth curves of individual rhizosphere isolates in 0.1× R2A medium. Download FIG S2, EPS file, 0.1 MB. Copyright © 2022 Coker et al. 2022 Coker et al. https://creativecommons.org/licenses/by/4.0/ This content is distributed under the terms of the Creative Commons Attribution 4.0 International license.

### α-diversity of the SynCom is maximized through adjustment of starting community ratios.

**FIG 3:**
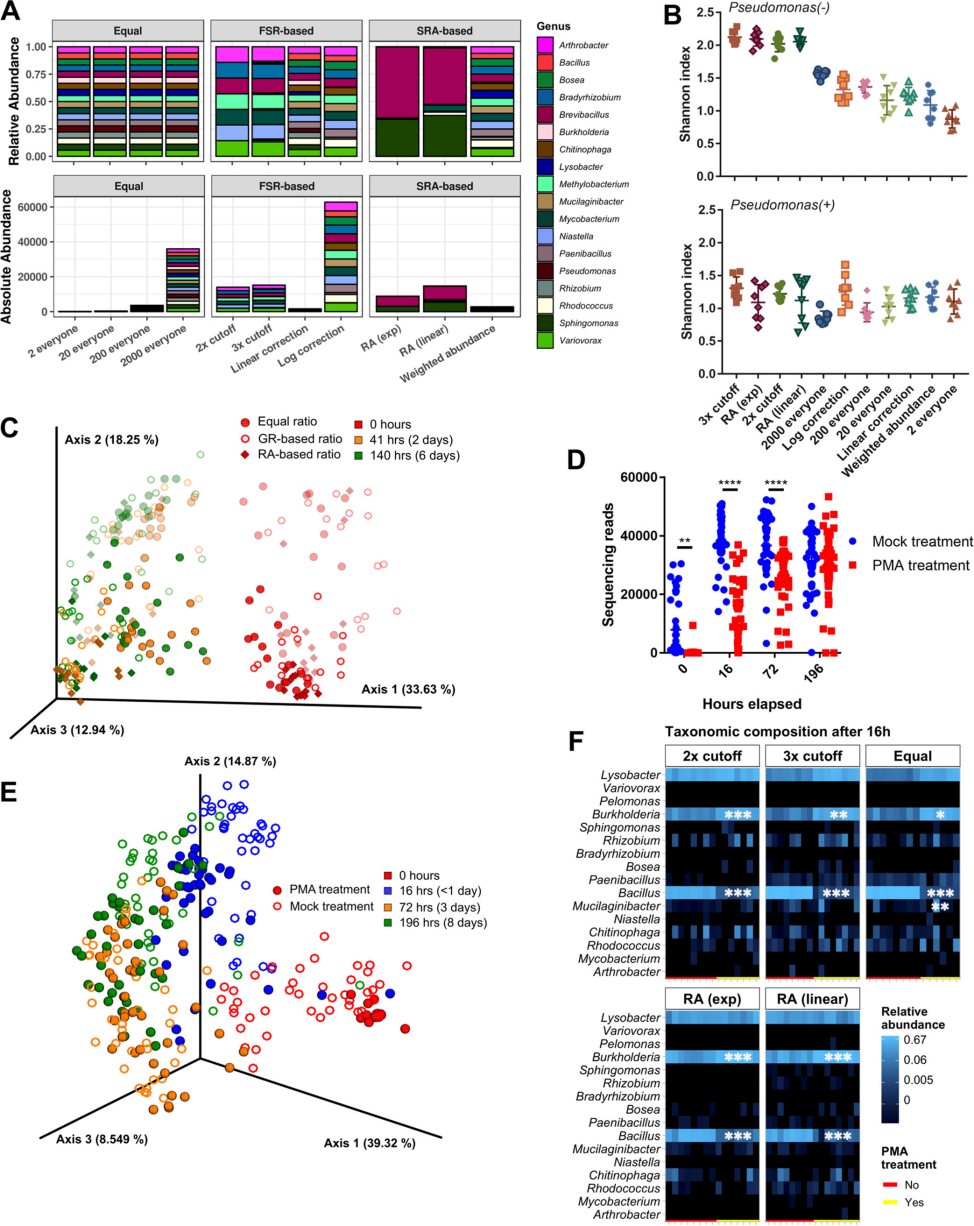
Community diversity with starting ratio adjustments and the removal of relic DNA. (A) Representation of the 11 community starting ratios used in this study, as both relative (top) and absolute (bottom) abundances. Descriptive names of the ratios are on the *x* axis. For equal communities, all organisms were added in equal but increasing amounts. The number refers to the number of drops released by the printer for each organism. For the FRA-based and SRA-based adjusted communities, the number of drops for each organism was calculated as shown in Tables S2 and S3. For the communities without Pseudomonas, Pseudomonas was not added. (B) The Shannon diversity index of each community ratio, 2 and 6 days combined. Communities without Pseudomonas are on the top, communities with Pseudomonas are on the bottom. Communities are shown in order of the decreasing average Shannon index for communities without Pseudomonas (*n* = 4 each). (C) PCA of the robust Aitchison distance between communities with different starting ratios. Symbols of communities with Pseudomonas have reduced opacity. GR, growth rate; RA, relative abundance. (D) Number of sequencing reads passing quality filtering per sample for the PMA-treatment and mock-treatment conditions (Student’s *t* test; **, *P* < 0.01; ****, *P* < 0.001). (E) PCA of Aitchison distance between the PMA-treatment and mock-treatment communities. (F) Heat map of taxonomic composition of the 5 community ratios after 16 h of growth, with the genera differentially abundant from the PMA treatment being marked with asterisks (DESeq2 Wald test, fitType = “parametric” with the BH FDR; *, *P*-adj < 0.05; **, *P*-adj < 0.01; ***, *P*-adj < 0.005). The PMA-treated (yellow) and mock-treated (red) communities are marked in the rug plot at the bottom of each heat map.

Next, we sought to maximize SynCom diversity through the adjustment of the community starting ratios, meaning that organisms were mixed in the starting community in ratios other than 1:1. We tested 11 different starting ratios with and without Pseudomonas (22 ratios total) (Fig. 3A). The exact calculations and ratios are provided in Tables S1 and 2. Briefly, the ratios were calculated based on the change in the relative abundance after 3 days of growth from an equally mixed inoculum. The starting relative abundance (SRA; relative abundance in the inoculum), final relative abundance (FRA; relative abundance after 3 days of growth), and FRA/SRA ratio (FSR) values were applied with various equations, with the goal being to design SynComs with high α-diversity. When designing the community compositions, we hypothesized that starting with smaller amounts of organisms with high FSR or FRA values and larger amounts of organisms with low SRA values would increase the α-diversity (“FSR-based” and “SRA-based” compositions) (Fig. 3A). We also included 4 compositions with different starting amounts of an equally mixed community (Fig. 3A, “Equal” compositions). For this study, two identical 96-well plates were assembled simultaneously with the picoliter printer and were allowed to grow for 2 and 6 days, respectively.

10.1128/msystems.00951-22.2 TABLE S2 Relative abundance values of organisms from an equally mixed community. Download Table S2, DOCX file, 0.2 MB. Copyright © 2022 Coker et al. 2022 Coker et al. https://creativecommons.org/licenses/by/4.0/ This content is distributed under the terms of the Creative Commons Attribution 4.0 International license.

In general, SynComs containing Pseudomonas grew to slightly higher OD_600_ values than did communities without Pseudomonas (Fig. S3A and B). The α-diversity, as measured by the Shannon index, was highest in the following SynComs without Pseudomonas: 2× cutoff, in which organisms with FSR < 0.05 received 2,000 drops from the starting isolate culture, whereas organisms with FSR > 0.5 received 2 drops; 3× cutoff, in which organisms with FSR < 0.05, FSR between 0.05 to 1, and FSR > 1 received 2000, 200, and 2 drops, respectively; relative abundance (RA) (exp), in which the number of drops decreases exponentially with the FRA; and RA (linear), in which the number of drops decreases linearly with the FRA (Fig. 3B). The α-diversity metric of Pielou’s evenness, calculated as the Shannon index divided by the natural log of the species richness, showed the same pattern, although with a smaller magnitude of difference (Fig. S3C and D). This indicates that both species richness and evenness drive the differences between SynComs. The analysis of the robust Aitchison distance, a metric of β-diversity, showed that the 2-day and 6-day communities were significantly different from the starting communities (pairwise PERMANOVA with the BH FDR correction, *P* = 0.0015) (Fig. 3C). The SynComs also separated between those with and without Pseudomonas (pairwise PERMANOVA with the BH FDR correction, *P* = 0.001).

10.1128/msystems.00951-22.6 FIG S3 Growth curves and evenness of communities with adjusted starting ratios. Community growth was monitored via the OD_600_ for 6 days. (A) Growth of communities without Pseudomonas simiae. (B) Growth of communities with Pseudomonas simiae. (C) Pielou’s evenness of communities without Pseudomonas simiae. (D) Pielou’s evenness of communities with Pseudomonas simiae. (E) Log-fold-change in *Burkholderia*, *Lysobacter*, *Chitinophaga*, and *Bacillus* between the 3× cutoff community samples on day 6 compared to those on day 2. The no spike-in samples are the log-fold-changes of the direct relative abundances. The spike-in samples are the log-fold-changes of the ratios of the indicated taxa to a standard amount of E. coli DNA spike-in. Download FIG S3, EPS file, 0.6 MB. Copyright © 2022 Coker et al. 2022 Coker et al. https://creativecommons.org/licenses/by/4.0/ This content is distributed under the terms of the Creative Commons Attribution 4.0 International license.

A recognized limitation of metagenome sequencing is the lack of information regarding absolute abundance levels in the community (41, 42). As such, the relative abundance of an organism could conceivably increase between conditions, even if its absolute abundance decreases, or *vice versa*. Comparing the change in organism ratios between samples (e.g., comparing the Taxa A/Taxa B ratio of sample 1 to the Taxa A/Taxa B ratio of sample 2) allows for the drawing of accurate conclusions regarding the changes in abundance, relative to other organisms in the community (43). To examine whether the relative abundance changes in the 3× cutoff SynCom accurately reflected the shifts in community proportions, we resequenced 4 samples of the 3× cutoff SynCom with a set amount of Escherichia coli DNA added as a spike-in. Then, we calculated the ratio of the four most abundant organisms (*Lysobacter*, *Burkholderia*, *Chitinophaga*, *Bacillus*) to E. coli and examined the log-fold change in the ratio between day 2 and day 6 of community growth. This ratio log-fold change was then compared to the relative abundance log-fold change of these organisms in the original sequencing data without the E. coli spike-in. The observed log-fold change values were similar with and without the spike-in (Fig. S3E), providing reassurance that the observed changes in the relative abundance are accurately reflecting the shifts in the community composition.

### Extracellular DNA affects community composition in early time points.

A well-known issue with DNA-based analysis is the inability to distinguish DNA from dead cells or other extracellular sources (“relic DNA”) from live cell DNA after sequencing (44–46). To determine the effect of relic DNA on our SynCom samples, we compared untreated communities to communities treated with propidium monoazide (PMA) to remove extracellular DNA prior to sequencing (46). The SynComs with the five highest α-diversity values from Fig. 3B were chosen to examine the effect of relic DNA. Four identical plates were prepared with the picoliter printer, with plates collected at time points 0, 16, 72, and 196 h. The overall community growth was not significantly different between SynComs (Fig. S4A and B).

10.1128/msystems.00951-22.7 FIG S4 Growth curves and taxonomic composition of PMA-treated and mock-treated communities. Community growth was monitored via the OD_600_ for 8 days (*n* = 4 for each condition). (A) Growth of communities that were not treated with PMA before sample collection. (B) Growth of communities treated with PMA before sample collection. (C and D) Heat map of taxonomic composition after 72 (C) or 196 (D) hours of growth, with all community ratios combined and with genera that are differentially abundant from those of the PMA-treatment being marked with asterisks (DESeq2 Wald test, fitType = “parametric” with the BH FDR; *, *P*-adj < 0.05; **, *P*-adj < 0.01; ***, *P*-adj < 0.005). PMA-treated (yellow) and mock-treated (red) communities are marked in the rug plot at the bottom of each heat map. Download FIG S4, EPS file, 1.8 MB. Copyright © 2022 Coker et al. 2022 Coker et al. https://creativecommons.org/licenses/by/4.0/ This content is distributed under the terms of the Creative Commons Attribution 4.0 International license.

16S rRNA gene sequencing of PMA-treated communities showed significantly fewer reads passing quality filtration at the 0, 16, and 72 h time points, compared to mock-treatment communities, although the gap decreased as time increased (Student’s *t* test; **, *P* < 0.01; ***, *P* < 0.001) (Fig. 3D). No difference was seen in the number of reads between the mock-treated and PMA-treated communities at 196 h. A PCA of the Aitchison distance between the SynComs showed a separation between the 0 h time point and the other time points (Fig. 3E). The PMA-treated samples were significantly different from the mock-treated samples at 0, 16, and 196 h but not at the 72-h time point (pairwise PERMANOVA with the BH FDR correction, *P* = 0.001 to 0.005). A taxonomic analysis showed significant differences in relative abundance between the PMA-treated and mock-treated communities at the 16 h time point (Wald test with the BH FDR; *, *P* < 0.05; **, *P* < 0.01; ***, *P* < 0.005) (Fig. 3F). For all of the SynCom ratios tested, PMA treatment significantly decreased the *Bacillus* relative abundance and increased the *Burkholderia* relative abundance. This was not observed at the 72 h time point, although it was seen to a lesser extent in the 196 h time point (Fig. S4C–D). This suggests that many of the *Bacillus* reads detected in the mock-treatment samples before 24 h could come from nonviable cells (presumably either spores or dead cells), whereas this is less likely to be the case after 24 h.

### SynCom diversity dynamics are driven by the presence of a few taxa.

**FIG 4:**
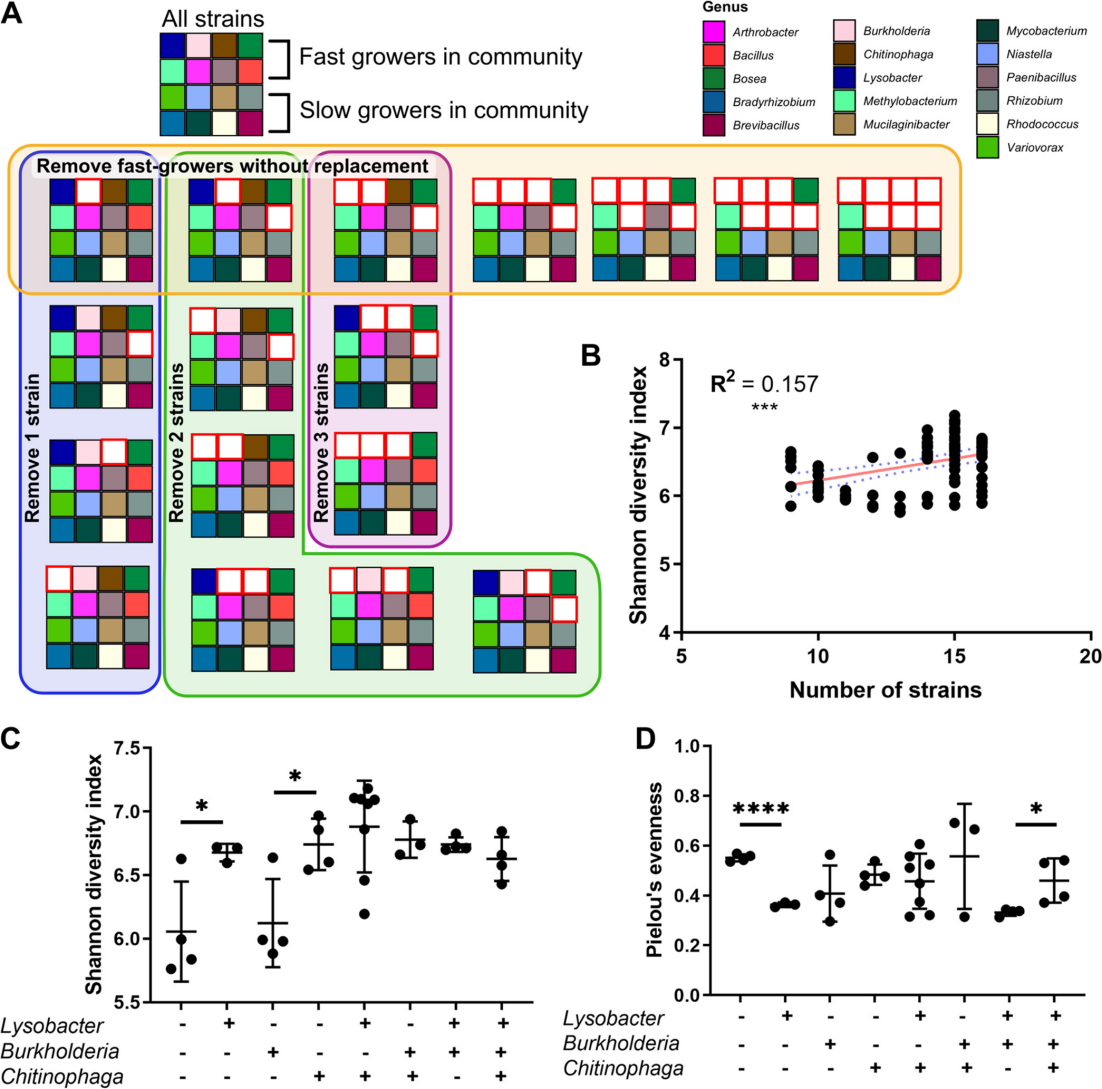
(A) Schematic of 18 tested combinations of the 3× cutoff community, with each large square representing a different combination. Each small colored square represents an individual strain (see the bottom right legend). White squares with a red border indicate that the organism in that position was not included in that combination. For this experiment, the strains were divided into fast-growing and slow-growing strains as indicated. (B) The number of strains in each community combination plotted against the Shannon diversity index (*n* = 3 to 8 per combination). A linear regression trendline and a 95% confidence interval are shown on the plot in red and blue, respectively. Spearman’s correlation coefficient is reported on the plot (***, *P* < 0.001). (C) The Shannon diversity index for combinations with 13 or more strains. The communities are divided into groups based on the presence/absence of *Lysobacter*, *Burkholderia*, and *Chitinophaga* (Student’s *t* test; *, *P* < 0.05). (D) Pielou’s evenness for combinations with 13 or more strains (Student’s *t* test, *, *P* < 0.05; ****, *P* < 0.001).

After determining that the highest α-diversity was observed in the 3× cutoff SynCom composition, we sought to determine whether the presence of specific taxa was required to generate this high-diversity community. For example, would there be a taxon or a group of taxa whose removal caused SynCom diversity to decrease sharply? To address this question, we started with the 3× cutoff SynCom and removed combinations of one or more organisms from the starting community. The absolute number of drops for the remaining organisms was left the same. *Sphingomonas* was not included because it was not seen to persist in the SynCom in any of the previous experiments. We tested a total of 18 combinations within the 3× cutoff SynCom (Fig. 4A). We compared the composition of the SynCom with 16 strains to the communities with only “fast-growing” strains (strains that received 2 drops during community assembly), only “slow-growing” strains (strains that received 2,000 drops during community assembly), combinations that removed 1, 2, or 3 strains at a time, and that or removed the fast-growing strains one-by-one without replacement. The growth curves for the communities are shown in Fig. S5A–E.

10.1128/msystems.00951-22.8 FIG S5 Growth curves and α-diversity of the modified 3× cutoff community compositions. (A–E) Community growth was monitored via the OD_600_ for 6 days (*n* = 4 for each condition). The 16-strain community and negative-control are included on all plots. (A) Growth of communities with all strains, only the “fast-growers” (received 2 drops in the community), the “middle-growers” (200 drops), or the “slow-growers” (2,000 drops). (B) Growth of communities removing the fast-growers, without replacement. Each community is missing the organisms removed in previous iterations as well as the organism directly listed. (C) Growth of communities removing individual fast-growers. (D) Growth of communities removing 2 fast-growers. (E) Growth of communities removing 3 fast-growers. (F) The number of strains in each community combination plotted against Pielou’s evenness (*n* = 3 to 8 per combination). A linear regression trendline and a 95% confidence interval are shown on the plot in red and blue, respectively. Spearman’s correlation coefficient is reported on the plot (value not significant). Download FIG S5, EPS file, 0.7 MB. Copyright © 2022 Coker et al. 2022 Coker et al. https://creativecommons.org/licenses/by/4.0/ This content is distributed under the terms of the Creative Commons Attribution 4.0 International license.

First, we examined the effect of the total number of strains on SynCom α-diversity, as measured by Shannon diversity index and Pielou’s evenness. There was a statistically significant but weak positive correlation between the number of strains in the starting community and the final Shannon diversity (Spearman’s correlation coefficient, R^2^ = 0.157, *P* = 0.002) (Fig. 4B). This correlation disappeared when examining Pielou’s evenness (Fig. S5F). However, we were most interested in the SynCom combinations that deviated from the linear regression trendline shown in Fig. 4B. These communities displayed a level of diversity that cannot be explained by the increased number of inoculated organisms. Therefore, we analyzed the composition of the SynComs with 13 or more organisms for patterns that could explain the large differences in α-diversity between communities. Each of these SynComs contained a “base community” of 13 organisms with additional combinations of the fast-growing *Lysobacter*, *Burkholderia*, and *Chitinophaga* strains (*x* axis) (Fig. 4C–D).

The Shannon diversity index, which incorporates both species richness and evenness, varied significantly depending on the combination of 3 organisms present in the SynCom. The addition of *Lysobacter* or *Chitinophaga* to the base community increased the Shannon diversity, but the addition of *Burkholderia* did not (Student’s *t* test, *, *P* < 0.05) (Fig. 4C). Adding both *Lysobacter* and *Chitinophaga* did not significantly change the Shannon diversity or the evenness over adding one of those organisms. Additionally, adding *Burkholderia* with *Lysobacter* or *Chitinophaga* did not reduce the Shannon diversity in the way that *Burkholderia* alone did.

Pielou’s evenness, which examines community evenness but not species richness, showed that the addition of *Lysobacter* to the base community significantly decreased evenness (Student’s *t* test, *, *P* < 0.05; ****, *P* < 0.001) (Fig. 4D). This contrasts with the observed increase in Shannon diversity in this community. Adding *Chitinophaga* or *Bukholderia* individually also resulted in decreased evenness, although this change was not statistically significant. Adding *Lysobacter* in the presence of *Burkholderia* still caused a highly significant decrease in evenness. However, adding all three organisms *Lysobacter*, *Burkholderia*, and *Chitinophaga* resulted in a significant increase in evenness.

### The SynCom is able to colonize the rhizosphere in an EcoFAB system.

To further our goal of developing a template for a model rhizosphere microbial community, we integrated our SynCom with the EcoFAB device (https://eco-fab.org/), an existing system developed for reproducible studies with plants (16, 18, 38). The colonization of plants in EcoFAB devices by the rhizosphere isolates would show that these SynComs can easily be transferred to a current plant-microbiome system. To investigate this, sterile *Brachypodium distachyon* Bd21-3 seedlings were transferred into the EcoFAB device at 3 days after germination. Then, 12-day-old plants were inoculated with an equally mixed SynCom, either with or without Pseudomonas, and were allowed to grow for 7 days (*n* = 4 to 5). The rhizosphere community composition was then assessed with 16S sequencing and was compared to the original inoculant.

**FIG 5:**
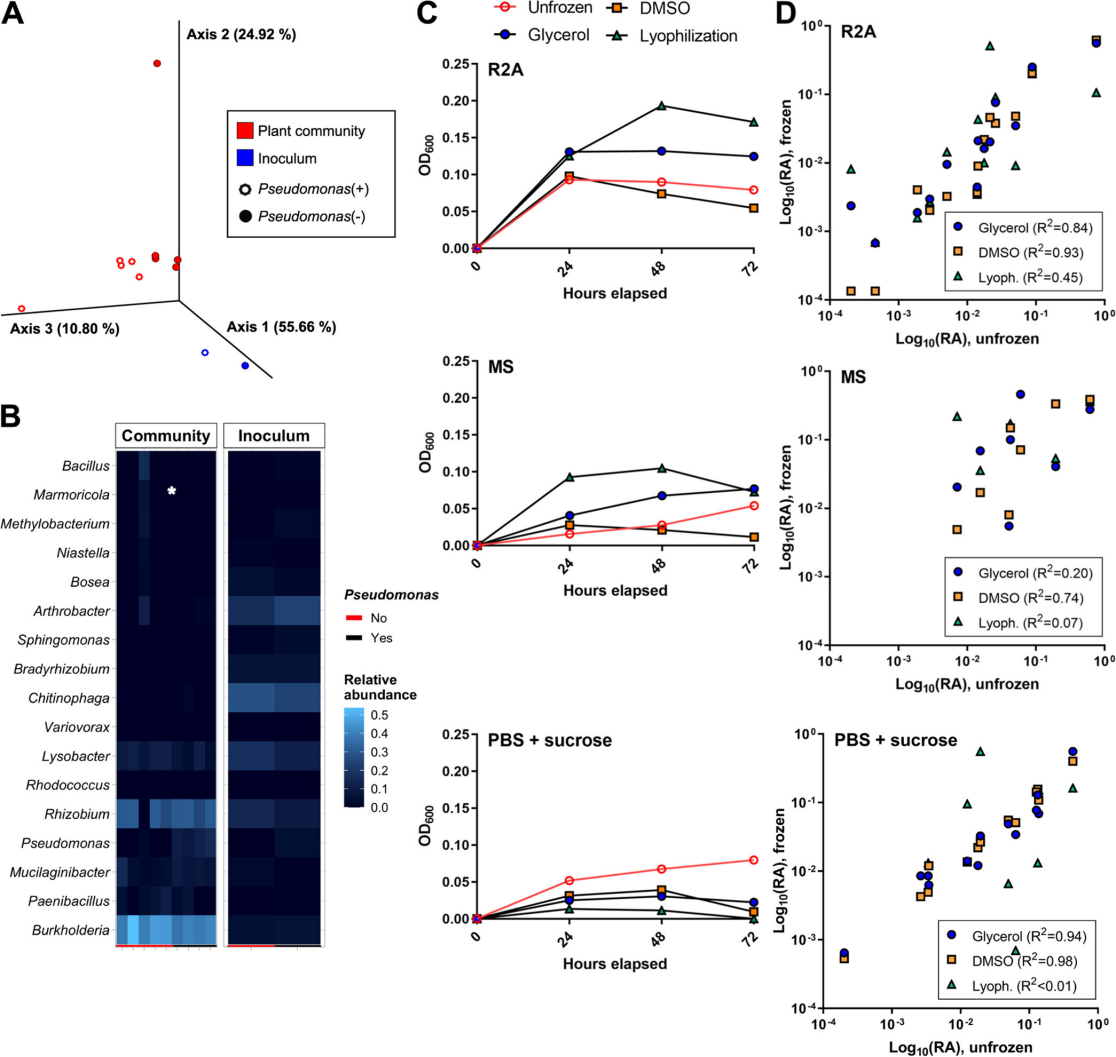
Community growth and composition with plant colonization and following cryopreservation. (A and B) An equally mixed community was inoculated onto 12-day-old *Brachypodium* plants, with or without Pseudomonas, and was allowed to grow for 7 days. (A) PCoA of the Bray-Curtis distances between the rhizosphere communities grown on plants for 7 days (*n* = 5 each) and the original inoculant (*n* = 1). (B) Relative abundance heat map of the starting inoculum and the rhizosphere communities grown on plants for 7 days. The rug plot indicates the presence (black) or absence (red) of Pseudomonas in the inoculant. Differential abundances between communities with and without Pseudomonas are marked with asterisks (DESeq2, Wald test, fitType = “parametric” with the BH FDR; *, *P* < 0.05). (C and D) An equally mixed community was preserved with 20% glycerol, 20% DMSO, or lyophilization and was regrown in R2A medium (left panel), MS medium (middle panel), or PBS with 10% sucrose (right panel). The growth and composition were compared to a community that was not frozen. (C) Community growth measured through the OD_600_. The unfrozen control community is shown in red. (*n* = 1) (D) A comparison of the log_10_ (relative abundance) from the 16S sequencing between the frozen and unfrozen communities for each cryopreservation method. Each point represents the log_10_ (relative abundance) of an individual genus in the frozen versus unfrozen community. Pearson’s correlation coefficient is reported for each comparison.

The SynComs grown on plants were significantly different from the original inoculant, as determined by the Bray-Curtis distance (pairwise PERMANOVA with the BH FDR correction, *P* = 0.036) (Fig. 5A). SynComs with Pseudomonas were not significantly different from communities without Pseudomonas. This was confirmed by comparing the relative abundance in SynComs with and without Pseudomonas, which showed only one significantly different, low-abundance organism (Wald test with the BH FDR, *, *P* < 0.05) (Fig. 5B). However, comparisons between plant communities and the inoculant show obvious changes in composition after 7 days on the plant, although significance testing could not be done due to the single batch of inoculant. *Burkholderia*, *Rhizobium*, and *Mucilaginibacter* increased in relative abundance, while several other organisms decreased in relative abundance. The increase in *Burkholderia* mirrors the presence of *Burkholderia* in the *in vitro* SynComs.

### Cryopreservation allows for SynCom regrowth that recapitulates the original community composition.

To enable collaborative and comparable microbiome research, any synthetic community must grow and act reproducibly between different researchers and research institutions. We sought to determine which method of cryopreservation would best preserve community fidelity to an unfrozen community in order to facilitate the distribution of this synthetic rhizosphere community to other research groups. We tested the growth and composition of an equally mixed SynCom, following three methods of cryopreservation, compared to an unfrozen control SynCom. We chose to test the cryopreservation methods of lyophilization, glycerol, and DMSO, as these are three of the most widely used methods in microbiology research. Lyophilization is typically preferred for long-term storage, but it is known to create viability issues due to the stress cells experience during vacuum desiccation (47, 48). Within a mixed community, this could conceivably result in viability differences between species. The cryopreserving agents DMSO and glycerol exert less stress on cells but have various levels of cell penetration between different cell types (49). Therefore, we sought to determine which method would most closely resemble an unfrozen community after thawing and reconstitution. The unfrozen SynCom was allowed to grow for 72 h in three types of media (R2A media, MS media, or PBS with 10% sucrose [the lyophilization medium]). The cryopreserved SynComs were frozen at −80°C for 3 days and were then thawed and subsequently grown for 3 days in the same media types as the unfrozen community.

The community growth was the highest in the R2A medium and the lowest in the PBS with 10% sucrose medium, regardless of the cryopreservation method (Fig. 5C). A comparison of the log-transformed relative abundance values from 16S sequencing showed large differences in community composition between the cryopreservation methods (Fig. 5D). Lyophilization consistently produced the lowest Pearson’s coefficient of determination (R^2^ value) between the frozen and unfrozen communities in all media. Glycerol and DMSO showed similar high coefficients in the R2A and PBS with 10% sucrose media, although the DMSO coefficient was much higher than that of glycerol in the MS medium (R^2^ = 0.74, compared to R^2^ = 0.20).

## DISCUSSION

In this study, we sought to develop a method to assemble and manipulate a synthetic rhizosphere community as a model for microbiome research while maintaining high levels of community α-diversity. The goal was a SynCom that is diverse, reproducible, and easily shared between labs. To facilitate sharing with other research groups, all isolates have been submitted to the DSMZ strain collection. The DSMZ IDs are available in the Materials and Methods section. A detailed protocol for SynCom assembly, by hand and by machine, has also been provided in the Supplemental Material (Text S1). Furthermore, we investigated community cryopreservation methods to determine the optimal method. The three methods tested, namely, lyophilization, DMSO, and glycerol freezing, are popular methods that are common for microbial preservation, and their pros and cons have been studied extensively for individual organisms (49). However, to our knowledge, only one study has investigated cryopreservation in communities for simple 2-member and 3-member constructed microbiomes (50). Thus, our study is one of the first to examine the impact of preservation methods on a multimembered, synthetically constructed microbial community. Although the sequencing results indicated a high correlation between glycerol-frozen and DMSO-frozen SynComs and the unfrozen SynCom in PBS with 10% sucrose, the low OD_600_ values in this medium indicate that this fidelity is likely due to a lack of growth following thawing. Preservation with 20% glycerol and regrowth in an MS medium led to similar growth by OD_600_ to that of the unfrozen SynCom, but the sequencing results revealed a poor correlation. However, glycerol preservation and regrowth in 0.1× an R2A medium resulted in SynCom regrowth that closely recapitulated the unfrozen community in terms of both OD_600_ and community composition, based on sequencing results. The SynCom can therefore be directly revived from glycerol-preserved stocks, allowing it to be shared between multiple labs and obviating the need for repeated reconstruction.

To address issues of reproducibility, we investigated using a liquid-handling robot system to prepare the SynComs. The direct comparison of hand-assembled and machine-assembled SynComs showed that the machine-assembled communities generally have a lower level of dissimilarity than do the communities assembled by hand (Fig. 2B). Examining SynCom α-diversity (Fig. S1C), two of the four hand-assembly subjects produced communities with a similar standard deviation to that of the machine-assembled communities, indicating that SynComs can be assembled by hand with careful and precise pipetting. However, the other two subjects produced communities with a larger standard deviation, indicating the differences inherent between hand-assembly subjects. Therefore, these communities can be assembled by hand reproducibly in labs without a CellenONE or similar liquid-handling robot. However, machine assembly reduces this source of variability in SynCom production and facilitates high-throughput experiments.

The picoliter printer addresses the issue of inaccurate cell-number estimates using conventional light-scattering and turbidity/absorbance measurements. Cell clumping, biofilm formation, culture conditions, and cell shape/geometry can affect the cell-counts estimated from optical density-based techniques (51, 52). Efforts to correct for this by using universal calibration strategies have yielded some success but are highly specific to the cell-type being measured and can therefore become unreliable when working with different isolates with different cell shapes and properties, such as in synthetic community construction (53). We note that the picoliter printer’s ability to visualize cells in the drops prior to dispensing (Fig. 1B–D) circumvents these critical issues and obviates the need for cross-calibrating spectrophotometric instruments for every member of the community.

After establishing SynCom reproducibility, we next focused on SynCom diversity. Previous work has indicated that most microbial interactions in communities are competitive (54, 55). *In vitro* communities often lose α-diversity over time, presumably as dominant microbes outcompete less-abundant microbes. Other soil microbiome studies that started with a large number of community members (21, 30) reported a loss of detection of many organisms after the community was applied to plants. Of our 17 starting organisms (excluding Pseudomonas), 11 were found consistently throughout all experiments and time points. Pseudomonas simiae was excluded from our later SynCom experiments due to its tendency to proliferate rapidly in the community and decrease the overall α-diversity. The exact mechanisms driving the loss of community organisms are difficult to pinpoint in any study, often requiring multi-omic methods. Metagenomics can suggest the functional capacity of a community, but to draw accurate conclusions about the function of a community or an individual microbe, it must be accompanied by data on which genes are being expressed and which proteins are being translated. While these analyses are outside the scope of this study, they could be pursued to investigate the role of individual microbes within the community.

We were able to substantially increase SynCom α-diversity, in terms of both Shannon diversity and Pielou’s evenness, by adjusting the starting organism ratios based on the growth rates of the community members. The Shannon diversity index uses both the species richness and the community evenness to calculate α-diversity. The maximum Shannon diversity for an individual community is log(k), where k represents the species richness (i.e., the number of taxa). The maximum value is achieved when all taxa have equal abundance in the community. Pielou’s evenness is calculated by dividing the Shannon diversity index by the natural log of the species richness, resulting in a value between 0 and 1. An evenness of 1 indicates a community with equal relative abundance between all taxa.

Starting ratios containing orders of magnitude more of slower-growing organisms (2× cutoff and 3× cutoff) resulted in higher Shannon diversity and evenness values than did the 4 equally mixed conditions (Fig. 3B; Fig. S3D). The increases in α-diversity were seen even after 6 days of community growth. This indicates that SynCom diversity can be increased over the diversity seen in typical 1:1-ratio communities by determining the growth rate of each individual member and adjusting the starting ratio accordingly. As presented in this study, these ratios can also be determined through the calculation of relative abundance changes during the growth of an equally mixed community.

In our 3× cutoff SynCom without Pseudomonas, which displayed the highest α-diversity of the tested starting compositions, we further investigated the specific combination of organisms driving community diversity. We did not see a strong relationship between α-diversity and the total number of organisms in the starting SynCom (Fig. 4B; Fig. S5F). This finding shows that the identity of organisms in the community drives α-diversity, not merely the absolute number of organisms. Indeed, in SynComs with 13 or more organisms, we noticed a range of α-diversity. We therefore analyzed the changes in α-diversity with the presence or absence of specific fast-growing organisms in the community. Our results indicate that different combinations of these organisms within the 3× cutoff SynCom produce intriguing changes in the species richness and evenness of the final community. For example, the addition of *Lysobacter* significantly decreased evenness but significantly increased the Shannon diversity (Fig. 4C and D), indicating that *Lysobacter* must increase species richness. This change goes beyond the increase expected from adding *Lysobacter* itself because the addition of *Burkholderia* alone does not increase the Shannon diversity. A possible explanation for this is that *Lysobacter* enables the growth of other low-abundance organisms in the community. In another example, the addition of *Lysobacter*, *Burkholderia*, and *Chitinophaga* together did not result in Shannon diversity changes compared to the addition of *Lysobacter* and *Burkholderia*, but it did result in increased evenness. This would indicate that adding the three organisms together promotes a more compositionally balanced community without increasing species richness, possibly due to competition between these three fast-growing organisms.

To address the potential influence of relic DNA on our sequencing results, we tested the effect of PMA treatment on the SynComs with the highest α-diversity. Our results indicate that relic DNA can have a significant effect on sequencing results from <24 h of community growth. Significantly fewer quality sequencing reads were detected in the PMA-treated communities after 0, 16, and 72 h of growth. However, the taxonomy relative abundance values were similar from 72 h out to 196 h (8 days). This indicates that while relic DNA can significantly alter sequencing results in our system in short-term growth studies, this effect diminishes at later time points (between 24 and 72 h). Additionally, PMA can be used to estimate bacterial viability if enough sample is available. Numerous studies have applied PMA to bacterial isolates or communities to quantify the number of live and dead cells (45, 46, 56, 57). Therefore, the SynCom samples could be split into two portions, with one portion treated with PMA, and the number of cells could be compared between the portions via flow cytometry or PCR. This technique could be used to assess the community viability if the OD is not a suitable metric.

After investigating and optimizing α-diversity for our rhizosphere SynCom, we next sought to display the utility of this community in a controlled model microbiome system. We tested the SynCom colonization of the EcoFAB device, a system designed for reproducible plant-microbiome system studies. The community was tested in the EcoFAB, using the model monocot Brachypodium distachyon, a prominent grass across arid and semiarid fields that is also growing in importance as a promising model system for biofuel crops (38, 58). Understanding how this plant influences the prominent members of the soil rhizobium could reveal novel biomass production enhancement strategies. The synthetic community was able to colonize the rhizosphere of *Brachypodium distachyon* plants grown in the EcoFAB device and was significantly different from the original inoculant after 7 days of growth on the plant (Fig. 5A and B). The presence of Pseudomonas did not significantly change the community in the EcoFAB system, unlike what was seen in our *in vitro* SynCom. The high relative abundance of *Burkholderia* in the rhizosphere communities was similar to the levels of *Burkholderia* seen in the *in vitro* SynCom, whereas other organisms had different relative abundance levels. These differences were expected, given the addition of the *Brachypodium* plant, which produces factors that affect soil microbe growth and metabolism. Indeed, aspects of plant-associated microbiomes have been shown to change rapidly in the natural environment, based on climatic factors (59). Future studies will focus on improving the accuracy of the *in vitro* community by adding plant factors to the growth media. The SynCom can easily be used with other fabricated ecosystems lacking a tunable microbial component, including devices that look specifically at root morphological responses to microenvironmental changes, such as the RootChip (60), Dual-Flow-RootChip (61), RootArray (62), or even systems that incorporate gravitropic growth, such as the PlantChip (63).

Community reproducibility, diversity, and preservation are essential questions to be addressed in the development of reproducible microbiome model communities. In developing a defined and reproducible synthetic microbial community, accounting for various starting organism ratios and the ability to preserve communities for dissemination are key elements to aid in reproducible microbiome sciences. Additionally, we have shown that our SynCom can be used in EcoFAB devices to reproducibly study plant-microbe interactions in the rhizosphere. The methods and workflows developed here can be readily adapted for the design and study of other model communities and to standardize microbiome research.

## MATERIALS AND METHODS

### Isolate selection.

Isolates were selected from a collection obtained from the rhizosphere and soil surrounding a single switchgrass plant grown in marginal soils described elsewhere (64, 65). These isolates are novel strains from unknown species, and strain identifiers can be found in Table S1. The final 16-member community isolates and details on their isolation are available from the Leibniz Institute German Collection of Microorganisms and Cell Cultures GmbH (DSMZ) under accession numbers DSM 113524 (*Arthrobacter* OAP107), DSM 113628 (*Bosea* OAE506), DSM 113701 (*Bradyrhizobium* OAE829), DSM 113525 (*Brevibacillus* OAP136), DSM 113627 (*Burkholderia* OAS925), DSM 113563 (*Chitinophaga* OAE865), DSM 113522 (*Lysobacter* OAE881), DSM 114042 (*Marmoricola* OAE513), DSM 113562 (*Mucilaginibacter* OAE612), DSM 113602 (*Methylobacterium* OAE515), DSM 113539 (Mycobacterium OAE908), DSM 113593 (*Niastella* OAS944), DSM 113526 (*Paenibacillus* OAE614), DSM 113517 (*Rhizobium* OAE497), DSM 113518 (*Rhodococcus* OAS809), and DSM 113622 (*Variovorax* OAS795). *Sphingomonas* OAE905 was not included in the final community and was therefore not deposited in the public repository. Pseudomonas simiae WCS 417 was previously published (66).

### Soil isolate growth conditions.

Individual isolates were grown in 3 to 5 mL liquid cultures of 1× R2A medium (Teknova, cat number R0005) in 14 mL culture tubes in aerobic conditions at 30°C without shaking. The isolates were allowed to grow for 5 to 7 days before dilution for community generation. 0.2× and 0.1× media were made by diluting 1× medium with water purified by a Milli-Q water purification system and vacuum-filtering through a 0.22 μM filter. The growth curves for the individual isolates were conducted in 96-well plates. Isolates cultured in 1× R2A medium were diluted to a starting OD_600_ of 0.05 in 200 μL of 0.1× R2A. Sterile R2A medium served as the negative control.

### Synthetic community growth conditions.

Communities were grown in 200 μL of liquid R2A medium in 96-well plates in aerobic conditions at 30°C without shaking. To prevent condensation, each plate lid was coated with 3 mL of an aqueous solution with 20% ethanol and 0.01% Triton X-100 (Sigma, cat number X100-100ML). The excess liquid was removed after 30 s, and the lid was allowed to air dry for 30 min under a UV light for sterilization. To further prevent condensation, the plates were set on 4 100 mm diameter petri dishes (2 stacks of 2 dishes), each of which was filled with ~20 mL of water to generate a humid environment around the plates. To normalize the isolates and monitor the community growth, optical density readings at 600 nm were taken with a Molecular Devices SpectraMax M3 Multi-Mode Microplate Reader (VWR, cat number 89429-536).

### Synthetic community assembly using the CellenONE printer.

Communities were assembled using a SCIENION CellenONE machine (https://www.scienion.com/). Individual isolate cultures were OD_600_-normalized to 0.025 (after subtracting a media blank) and were then transferred from a 384-well or 96-well probe plate to a 96-well target plate using a CellenONE piezo dispense capillary (PDC) (size medium; Scienion, cat #, *P*-20-CM) with the droplet size set to 390 to 420 picoliters. The selection criteria were adjusted and visually confirmed to ensure that 2 to 3 cells were dispensed per drop (Fig. 1B–D). The number of drops per isolate for each community was programmed by hand, using the provided Scienion software (v1.92). The number of drops per organism for each ratio can be found in Table S3. Droplet integrity was confirmed before and after each isolate spotting run, using the droplet camera and automated droplet detection. The PDC was cleaned between isolates by flushing the PDC interior with 0.5 mL of water. 200 drops of R2A were added to the negative-control wells as the last step in each experimental setup to ensure that no contamination occurred due to the incomplete flushing of the PDC between isolates. For the community dynamics experiment, organisms receiving 2,000 drops were added to communities with a multichannel pipettor.

10.1128/msystems.00951-22.3 TABLE S3 Equations for starting community ratios and the number of CellenONE printer drops per organism. Download Table S3, DOCX file, 0.2 MB. Copyright © 2022 Coker et al. 2022 Coker et al. https://creativecommons.org/licenses/by/4.0/ This content is distributed under the terms of the Creative Commons Attribution 4.0 International license.

### Treatment with PMA to remove relic DNA.

PMA (Biotium, cat number 40013) was added to communities to a final concentration of 10 μM directly prior to sample collection (PMA-treatment), and 5 μL of water (mock treatment) was added to the control communities. Then, the communities were incubated in the dark for 5 min at room temperature, and were then placed <15cm from a direct fluorescent light source and incubated on ice for 30 min. The communities were then frozen at −20°C until processing for sequencing.

### Plant colonization experiment.

*Brachypodium distachyon* Bd21-3 seeds were dehusked, sterilized, and germinated on 0.1× Murashige and Skoog (MS) basal salt mixture M524 plates, pH 5.7 (Phyto Technology Laboratories) in a 250 μmol/m^2^ s^−1^ 16 h light/8 h dark regime at 24°C for 3 days. The EcoFABs were sterilized as previously described (18), and the seedlings were transferred to EcoFAB chambers filled with 0.1× MS at 3 days after germination. 12-day old plants were inoculated with an equally mixed community of 17 or 18 bacterial isolates, as described above. To mix the community, the OD_600_ of each isolate was measured, with the assumption that an OD_600_ of 1 is equal to ~10^9^ colony-forming unites (CFU)/mL. Isolates were combined at 10^5^ CFU/mL per isolate in the final EcoFAB volume. Plants were harvested 7 days after inoculation. Microbial communities were detached from the plant root by vortexing the root in 0.1 phosphate-buffered saline (PBS) for 10 min at maximum speed, followed by centrifugation at 10,000 × *g* at 6°C. DNA was extracted by using the Qiagen DNeasy PowerSoil Pro Kit according to the manufacturer’s instructions (cat number 47014).

### Community cryopreservation and regrowth.

All community members were OD_600_-normalized to 0.1 after 3 days of growth in 1× R2A and were mixed equally to a final estimated total CFU count of 7.2·10^8^ CFU. The community was then centrifuged (5000 × *g*, 5 min) and resuspended in 4 mL of 0.1× R2A medium. 250 μL of the community was inoculated into 4 mL of 0.1× R2A, MS medium (RPI, cat number M10200), or PBS + 10% sucrose (wt/vol) as the “unfrozen” control community. 500 μL of the community was mixed with 500 μL of either 40% glycerol, 40% DMSO, or PBS with 20% sucrose (wt/vol). The glycerol and DMSO stocks were frozen immediately at −80°C. The PBS with 10% sucrose stock was lyophilized on a Labconco FreeZone Plus Freeze Dry System (cat number 7386030) and then stored at −80°C. The stocks were thawed after 3 days, and 250 μL of stock were inoculated into the same 3 types of media as the unfrozen community. Samples from all communities were frozen at −20°C after 3 days of growth for 16S sequencing analysis.

### DNA extraction and sequencing.

DNA extracted with a kit was processed with the Qiagen DNeasy PowerSoil Pro Kit according to the manufacturer’s instructions (cat number 47014). DNA extracted by boiling was processed by thawing community samples, transferring 100 μL to a PCR plate, and heating the plate in a PCR machine at 100°C for 10 min. 5 μL of undiluted sample was used as the DNA input for the 16S rRNA gene amplicon library protocol. The 16S libraries for the cryopreservation, adjusted community ratios, PMA, and boil-extraction comparison experiments were prepared using 515F-806R primers according to the Earth Microbiome Project protocol (67) and were sequenced on an Illumina MiSeq platform with a paired-end 150 V2 kit as previously described (68, 69). 16S libraries for the community dynamics experiment were prepared using 341F-805R primers (F 5′-CCTACGGGNGGCWGCAG-3′ R 5′-GACTACHVGGGTATCTAATCC-3′) and were sequenced on an Illumina MiSeq platform with a paired-end 150 V2 kit. The 16S libraries for the plant experiments were prepared using 515F-806R primers and were sequenced on an Illumina NovaSeq platform with a paired-end 250 V2 kit. Shotgun metagenomics libraries for the human-assembled/machine-assembled experiment were prepared using 1 ng of DNA input and Nextera XT indexes and were sequenced on an Illumina MiSeq platform with a paired-end 150 V2 kit.

### 16S rRNA sequencing analysis and statistical analyses.

All of the 16S sequences were analyzed using QIIME2 (70) (v2020-11). Paired-end reads were joined using the “qiime vsearch join-pairs” command and were quality-filtered and denoised (using the default parameters) with Deblur (71). The reads were trimmed as appropriate for quality for each experiment (150 bp for the human-/machine-assembled, cryopreservation, adjusted community ratios, and PMA experiments; 200 bp for the plant experiment). The α-diversity and β-diversity values were calculated using the “qiime diversity” set of commands, with rarefaction used to normalize the data to an appropriate sampling depth. The robust Aitchison distance was calculated using the DEICODE plugin (72). Microbial taxonomy was assigned to the filtered sequences with the “qiime feature-classifier classify-sklearn” command, using a scikit-learn classifier created from a custom database of the 16S rRNA gene sequences for the isolates used in the study. Heat maps and relative abundance plots were generated using R (73) (v3.3.2) with the packages dplyr (74), phyloseq (75), ggplot2 (76), scales (77), and DESeq2 (78). β-diversity plots were generated using QIIME2. All other plots were generated using GraphPad Prism 7 software.

### E. coli DNA spike-in experiments.

Escherichia coli MG1655 was grown as an overnight culture in LB broth at 30°C. The culture was spun down as a pellet, and DNA was extracted using a Qiagen DNeasy PowerSoil Pro Kit according to the manufacturer’s instructions (cat number 47014). A known amount of E. coli DNA was added to the extracted DNA of samples from the 3× cutoff community such that the spike-in was 5% of the total DNA in the samples by weight (ng). The samples were then prepped for 16S rRNA sequencing as described above and were sequenced using same parameters as before. For the spike-in communities, the ratio of each organism to E. coli was calculated. As described above, this ratio will be the same, whether calculated with relative or absolute abundance (43). The fold change in the taxa/E. coli ratio (spike-in communities) or relative abundance (original communities) between day 6 and day 2 was calculated for each organism in each community.

### Shotgun metagenomics sequencing analysis.

Shotgun sequencing data were quality-filtered during adapter trimming with Trimmomatic (79) (v0.36), using the settings “ILLUMINACLIP:NexteraPE-PE.fa:2:30:10 LEADING:10 TRAILING:10 SLIDINGWINDOW:4:15 MINLEN:36”. The trimmed reads were aligned to a custom database of community strain genomes using bowtie2 (80) (v2.2.3), using the default settings. The α- and β-diversity were calculated in phyloseq. β-diversity plots were generated using phyloseq. All other plots were generated using GraphPad Prism 7 software.

10.1128/msystems.00951-22.9 TEXT S1 Step-by-step protocol for SynCom assembly with the CellenONE machine and by hand. Download Text S1, DOCX file, 0.02 MB. Copyright © 2022 Coker et al. 2022 Coker et al. https://creativecommons.org/licenses/by/4.0/ This content is distributed under the terms of the Creative Commons Attribution 4.0 International license.

### Data availability.

DNA sequences generated through this study are available on the NCBI Sequence Read Archive (PRJNA807292). All code used to process and analyze sequencing results can be accessed through Github at https://github.com/jkccoker/Soil_synthetic_community.
