# Supplementary material for: A Reproducible and Tunable Synthetic Soil Microbial Community Provides New Insights into Microbial Ecology
Source: mSystems. 2022 Dec 6;7(6):e00951-22. doi: 10.1128/msystems.00951-22 (PMC9765266; doi:10.1128/msystems.00951-22)
Supplement: TABLE S1 [file msystems.00951-22-s0006.docx]

| **Genus** | **Strain** | **Associated plants^a^** |
| --- | --- | --- |
| *Lysobacter* | OAE881 | Nicotiana tabacum L, tomato, pepper^1–3^ |
| *Burkholderia* | OAS925 | Zea mays L, Betula, Equisetum, Quercus, Senecio vulgaris, Triticum aestivum, Zantedeschia, Coffea, Saccharum officinarum, Zea mays L, Zea mays L, Lolium multiflorum, citrus, wheat^4–9^ |
| *Variovorax* | OAS795 | Citrus, maize, tomato, wheat^8,10,11^ |
| *Chitinophaga* | OAE865 | Tomato, Oryza sativa L, Cymbidium goeringii, ginseng^12–15^ |
| *Niastella* | OAS944 | Hibiscus syriacus L, persimmon tree, Populus euphratica, Korean ginseng^16–19^ |
| *Mucilaginibacter* | OAE612 | Gossypium hirsutum L, Angelica sinensis, ginseng, Dokdo Island (S Korea)^20–23^ |
| *Sphingomonas* | OAE905 | Citrus, wheat, Oryza sativa L^8–10,24,25^ |
| *Rhizobium* | OAE497 | Citrus, Oryza sativa, Dioscorea alata, Dioscorea esculenta^10,26,27^ |
| *Bradyrhizobium* | OAE829 | Citrus, Vaccinium angustifolium, wheat, Brazilian sugarcane^8,10,28,29^ |
| *Bosea* | OAE506 | Zea mays L, Cyperus rotundus L^30,31^ |
| *Methylobacterium* | OAE516 | Eucalyptus spp., Oryza sativa cv. Dongjin and Lycopersicon esculentum L. cv. Mairoku, maize^32–34^ |
| *Arthrobacter* | OAP107 | Ginkgo biloba L, Quercus ilex, Triticum aestivum L (wheat)^35–37^ |
| *Mycobacterium* | OAE908 | Soil from Haikou (China), tomato, Oryza sativa L. cv. Wusimi^38–40^ |
| *Rhodococcus* | OAS809 | Zea mays L, Oryza sativa L.^30,41^ |
| *Brevibacillus* | OAP136 | Zea mays L, Lolium perenne, Pinellia ternata, Nicotiana tabacum L, Gossypium hirsutum^30,42–45^ |
| *Paenibacillus* | OAE614 | Solanum lycopersicum, Oryza sativa L, wheat^46–48^ |
| *Bacillus* | OAE603 | Zea mays L, Triticum aestivum L (wheat), Lolium perenne, Nicotiana tabacum L^30,37,42,44^ |
| *Pseudomonas simiae* | WCS417 | Wheat, citrus, Zea mays L, Nicotiana tabacum L^9,10,30,44^ |

^a^References were found through PubMed searches (conducted on 8/17/2021) of “<genus> rhizosphere”, “<genus> soil isolation”, and/or “<genus> plant isolation”. Plant scientific names are listed when included in the references; otherwise plant common names are used.

**Supplemental Table 1 References**

1. Xiao, M. *et al.* Lysobacter tabacisoli sp. nov., isolated from rhizosphere soil of Nicotiana tabacum L. *International Journal of Systematic and Evolutionary Microbiology* **69**, 1875–1880 (2019).

2. Kim, S. J. *et al.* Lysobacter solanacearum sp. nov., isolated from rhizosphere of tomato. *International Journal of Systematic and Evolutionary Microbiology* **67**, 1102–1106 (2017).

3. Park, J. H., Kim, R., Aslam, Z., Jeon, C. O. & Chung, Y. R. Lysobacter capsici sp. nov., with antimicrobial activity, isolated from the rhizosphere of pepper, and emended description of the genus Lysobacter. *International Journal of Systematic and Evolutionary Microbiology* **58**, 387–392 (2008).

4. Richardson, J., Stead, D. E., Elphinstone, J. G. & Coutts, R. H. A. *Diversity of Burkholderia isolates from woodland rhizosphere environments*.

5. Caballero-Mellado, J., Martínez-Aguilar, L., Paredes-Valdez, G. & Estrada-de los Santos, P. Burkholderia unamae sp. nov., an N2-fixing rhizospheric and endophytic species. *International Journal of Systematic and Evolutionary Microbiology* **54**, 1165–1172 (2004).

6. Ramette, A., LiPuma, J. J. & Tiedje, J. M. Species abundance and diversity of Burkholderia cepacia complex in the environment. *Applied and Environmental Microbiology* **71**, 1193–1201 (2005).

7. Castanheira, N. *et al.* Plant growth-promoting Burkholderia species isolated from annual ryegrass in Portuguese soils. *Journal of Applied Microbiology* **120**, 724–739 (2016).

8. Schlatter, D. C., Yin, C., Hulbert, S. & Paulitz, T. C. Core Rhizosphere Microbiomes of Dryland Wheat Are Influenced by Location and Land Use History. (2020) doi:10.1128/AEM.

9. Dai, Y. *et al.* Wheat-associated microbiota and their correlation with stripe rust reaction. *Journal of Applied Microbiology* **128**, 544–555 (2020).

10. Xu, J. *et al.* The structure and function of the global citrus rhizosphere microbiome. *Nature Communications* **9**, (2018).

11. Gao, J. lian *et al.* Variovorax beijingensis sp. nov., a novel plant-associated bacterial species with plant growth-promoting potential isolated from different geographic regions of Beijing, China. *Systematic and Applied Microbiology* **43**, (2020).

12. Kim, S. J. *et al.* Chitinophaga rhizosphaerae sp. Nov., isolated from rhizosphere soil of a tomato plant. *International Journal of Systematic and Evolutionary Microbiology* **67**, 3435–3439 (2017).

13. Chung, E. J., Park, T. S., Jeon, C. O. & Chung, Y. R. Chitinophaga oryziterrae sp. nov., isolated from the rhizosphere soil of rice (Oryza sativa L.). *International Journal of Systematic and Evolutionary Microbiology* **62**, 3030–3035 (2012).

14. Li, L. *et al.* Chitinophaga cymbidii sp. nov., isolated from Cymbidium goeringii roots. *International Journal of Systematic and Evolutionary Microbiology* **63**, 1800–1804 (2013).

15. Lee, H. G. *et al.* Chitinophaga ginsengisegetis sp. nov. and Chitinophaga ginsengisoli sp. nov., isolated from soil of a ginseng field in South Korea. *International Journal of Systematic and Evolutionary Microbiology* **57**, 1396–1401 (2007).

16. Yan, Z. F. *et al.* Niastella hibisci sp. nov., isolated from rhizosphere soil of mugunghwa, the Korean national flower. *International Journal of Systematic and Evolutionary Microbiology* **66**, 5218–5222 (2016).

17. Akter, S., Park, J. H., Mizanur Rahman, M. & Huq, M. A. Niastella soli sp. Nov., isolated from rhizospheric soil of a persimmon tree. *International Journal of Systematic and Evolutionary Microbiology* **71**, (2021).

18. Zhang, K. *et al.* Niastella populi sp. nov., isolated from soil of Euphrates poplar (Populus euphratica) forest, and emended description of the genus Niastella. *International Journal of Systematic and Evolutionary Microbiology* **60**, 542–545 (2010).

19. Weon, H. Y. *et al.* Niastella koreensis gen. nov., sp. nov. and Niastella yeongjuensis sp. nov., novel members of the phylum Bacteroidetes, isolated from soil cultivated with Korean ginseng. *International Journal of Systematic and Evolutionary Microbiology* **56**, 1777–1782 (2006).

20. Madhaiyan, M. *et al.* Mucilaginibacter gossypii sp. nov. and Mucilaginibacter gossypiicola sp. nov., plant-growth-promoting bacteria isolated from cotton rhizosphere soils. *International Journal of Systematic and Evolutionary Microbiology* **60**, 2451–2457 (2010).

21. Lee, H. R., Han, S. I., Rhee, K. H. & Whang, K. S. Mucilaginibacter herbaticus sp. nov., isolated from the rhizosphere of the medicinal plant Angelica sinensis. *International Journal of Systematic and Evolutionary Microbiology* **63**, 2787–2793 (2013).

22. Ahn, J. H. *et al.* Mucilaginibacter ginsengisoli sp. Nov., Isolated from a ginseng-cultivated soil. *International Journal of Systematic and Evolutionary Microbiology* **65**, 3933–3937 (2015).

23. Li, W. *et al.* Mucilaginibacter segetis sp. nov., Isolated from Soil. *Current Microbiology* **78**, 2447–2454 (2021).

24. Menon, R. R. *et al.* Sphingomonas pokkalii sp. nov., a novel plant associated rhizobacterium isolated from a saline tolerant pokkali rice and its draft genome analysis. *Systematic and Applied Microbiology* **42**, 334–342 (2019).

25. Chung, E. J. *et al.* Sphingomonas oryziterrae sp. nov. and sphingomonas jinjuensis sp. nov. isolated from rhizosphere soil of rice (oryza sativa l.). *International Journal of Systematic and Evolutionary Microbiology* **61**, 2389–2394 (2011).

26. Zhao, J. J. *et al.* Rhizobium oryziradicis sp. Nov., isolated from rice roots. *International Journal of Systematic and Evolutionary Microbiology* vol. 67 963–968 (2017).

27. Ouyabe, M. *et al.* Rhizobium dioscoreae sp. Nov., a plant growth-promoting bacterium isolated from yam (dioscorea species). *International Journal of Systematic and Evolutionary Microbiology* **70**, 5054–5062 (2020).

28. Morvan, S., Meglouli, H., Lounès-Hadj Sahraoui, A. & Hijri, M. Into the wild blueberry (Vaccinium angustifolium) rhizosphere microbiota. *Environmental Microbiology* **22**, 3803–3822 (2020).

29. de Alencar Menezes Júnior, I., Feitosa de Matos, G., Moura de Freitas, K., da Conceição Jesus, E. & Rouws, L. F. M. Occurrence of diverse Bradyrhizobium spp. in roots and rhizospheres of two commercial Brazilian sugarcane cultivars. *Brazilian Journal of Microbiology* **50**, 759–767 (2019).

30. Qaisrani, M. M. *et al.* A comparative study of bacterial diversity based on culturable and culture-independent techniques in the rhizosphere of maize (Zea mays L.). *Saudi Journal of Biological Sciences* **26**, 1344–1351 (2019).

31. Jurelevicius, D., Korenblum, E., Casella, R., Vital, R. L. & Seldin, L. Polyphasic analysis of the bacterial community in the rhizosphere and roots of Cyperus rotundus L. grown in a petroleum-contaminated soil. *Journal of Microbiology and Biotechnology* **20**, 862–870 (2010).

32. Andreote, F. D. *et al.* Culture-independent assessment of rhizobiales-related alphaproteobacteria and the diversity of Methylobacterium in the rhizosphere and rhizoplane of transgenic eucalyptus. *Microbial Ecology* **57**, 82–93 (2009).

33. Poonguzhali, S., Madhaiyan, M., Yim, W. J., Kim, K. A. & Sa, T. M. Colonization pattern of plant root and leaf surfaces visualized by use of green-fluorescent-marked strain of Methylobacterium suomiense and its persistence in rhizosphere. *Applied Microbiology and Biotechnology* **78**, 1033–1043 (2008).

34. Schmalenberger, A. & Tebbe, C. C. Bacterial community composition in the rhizosphere of a transgenic, herbicide-resistant maize (Zea mays) and comparison to its non-transgenic cultivar Bosphore. *FEMS Microbiology Ecology* **40**, 29–37 (2006).

35. Cheng, J. *et al.* Arthrobacter ginkgonis sp. Nov., an actinomycete isolated from rhizosphere of Ginkgo biloba L. *International Journal of Systematic and Evolutionary Microbiology* **67**, 319–324 (2017).

36. Fernández-González, A. J. *et al.* The rhizosphere microbiome of burned holm-oak: Potential role of the genus Arthrobacter in the recovery of burned soils. *Scientific Reports* **7**, (2017).

37. Upadhyay, S. K., Singh, D. P. & Saikia, R. Genetic Diversity of Plant Growth Promoting Rhizobacteria Isolated from Rhizospheric Soil of Wheat under Saline Condition. *Current Microbiology* **59**, 489–496 (2009).

38. Zhang, Y., Zhang, J., Fang, C., Pang, H. & Fan, J. Mycobacterium litorale sp. nov., a rapidly growing mycobacterium from soil. *International Journal of Systematic and Evolutionary Microbiology* **62**, 1204–1207 (2012).

39. Bouam, A., Armstrong, N., Levasseur, A. & Drancourt, M. Mycobacterium terramassiliense, Mycobacterium rhizamassiliense and Mycobacterium numidiamassiliense sp. nov., three new Mycobacterium simiae complex species cultured from plant roots. *Scientific Reports* **8**, (2018).

40. Wang, W., Zhai, Y., Cao, L., Tan, H. & Zhang, R. Illumina-based analysis of core actinobacteriome in roots, stems, and grains of rice. *Microbiological Research* **190**, 12–18 (2016).

41. Li, C. *et al.* Rhodococcus oryzae sp. nov., a novel actinobacterium isolated from rhizosphere soil of rice (Oryza sativa L.). *International Journal of Systematic and Evolutionary Microbiology* **70**, 3300–3308 (2020).

42. Li, Z., Song, C., de Jong, A. & Kuipers, O. P. Draft Genome Sequences of Six Bacillus Strains and One Brevibacillus Strain Isolated from the Rhizosphere of Perennial Ryegrass (Lolium perenne). (2019) doi:10.1128/MRA.

43. Sheng, M. *et al.* Siderophore Production by Rhizosphere Biological Control Bacteria Brevibacillus brevis GZDF3 of Pinellia ternata and Its Antifungal Effects on Candida albicans. *Journal of Microbiology and Biotechnology* **30**, 689–699 (2020).

44. Jin, F. *et al.* Genetic diversity and phylogeny of antagonistic bacteria against phytophthora nicotianae isolated from tobacco Rhizosphere. *International Journal of Molecular Sciences* **12**, 3055–3071 (2011).

45. Nehra, V., Saharan, B. S. & Choudhary, M. Evaluation of Brevibacillus brevis as a potential plant growth promoting rhizobacteria for cotton (Gossypium hirsutum) crop. *SpringerPlus* **5**, (2016).

46. Lee, S. A. *et al.* Paenibacillus lycopersici sp. nov. and Paenibacillus rhizovicinus sp. nov., isolated from the rhizosphere of tomato (Solanum lycopersicum). *Journal of Microbiology* **58**, 832–840 (2020).

47. Zhang, J. *et al.* Paenibacillus oryzisoli sp. nov., isolated from the rhizosphere of rice. *Antonie van Leeuwenhoek, International Journal of General and Molecular Microbiology* **110**, 69–75 (2017).

48. Ran, J. *et al.* Characterization of a novel antifungal protein produced by Paenibacillus polymyxa isolated from the wheat rhizosphere. *Journal of the Science of Food and Agriculture* **101**, 1901–1909 (2021).
