# Supplementary material for: A Reproducible and Tunable Synthetic Soil Microbial Community Provides New Insights into Microbial Ecology
Source: mSystems. 2022 Dec 6;7(6):e00951-22. doi: 10.1128/msystems.00951-22 (PMC9765266; doi:10.1128/msystems.00951-22)
Supplement: TABLE S2 [file msystems.00951-22-s0007.docx]

|  | **SRA^a^** | **FRA^b^** | **F/S ratio (FRA/SRA)^c^** |
| --- | --- | --- | --- |
| *Lysobacter* OAE881 | 0.029507 | 0.160573 | 5.44179057 |
| *Pseudomonas simiae* WCS417 | 0.139359 | 0.570065 | 4.09063447 |
| *Sphingomonas* OAE905 | 0.000018 | 0.000053 | 2.8754562 |
| *Burkholderia* OAS925 | 0.044979 | 0.117325 | 2.60846168 |
| *Rhizobium* OAE497 | 0.001012 | 0.001477 | 1.45909576 |
| *Bacillus* OAE603 | 0.363800 | 0.137535 | 0.37805086 |
| *Chitinophaga* OAE865 | 0.044788 | 0.007738 | 0.17275925 |
| *Mucilaginibacter* OAE612 | 0.023927 | 0.002400 | 0.10030911 |
| *Bosea* OAE506 | 0.001536 | 0.000085 | 0.0554598 |
| *Rhodococcus* OAS809 | 0.000219 | 0.000012 | 0.05523912 |
| *Paenibacillus* OAE614 | 0.047480 | 0.002502 | 0.05269173 |
| *Niastella* OAS944 | 0.016974 | 0.000045 | 0.0026626 |
| *Variovorax* OAS795 | 0.024828 | 0.000059 | 0.00237307 |
| *Arthrobacter* OAP107 | 0.250202 | 0.000049 | 0.0001968 |
| *Bradyrhizobium* OAE829 | 0.001405 | 0.000000 | 0 |
| *Methylobacterium* OAE516 | 0.001405 | 0.000000 | 0 |
| *Mycobacterium* OAE908 | 0.000120 | 0.000000 | 0 |
| *Brevibacillus* OAP136 | 0.000000 | 0.000019 | 0 |

^a^SRA = starting relative abundance; RA reported by 16S sequencing at time 0 of an equally-mixed community.

^b^FRA = final relative abundance; RA reported by 16S sequencing after 3 days growth of an equally-mixed community.

^c^F/S ratio = fold-change in relative abundance between time 0 and 3 days, calculated as FRA / SRA
