# Supplementary material for: A Reproducible and Tunable Synthetic Soil Microbial Community Provides New Insights into Microbial Ecology
Source: mSystems. 2022 Dec 6;7(6):e00951-22. doi: 10.1128/msystems.00951-22 (PMC9765266; doi:10.1128/msystems.00951-22)
Supplement: TABLE S3 [file msystems.00951-22-s0008.docx]

|  | **2 everyone** | **20 everyone** | **200 everyone** | **2000 everyone** | **2x cutoff** | **3x cutoff** | **Linear correction** | **Log correction** | **RA (exp)** | **RA (linear)** | **Weighted abundance** |
| --- | --- | --- | --- | --- | --- | --- | --- | --- | --- | --- | --- |
| **Equation^a^** | 2 drops | 20  drops | 200 drops | 2000 drops | 2 or 2000 drops | 2, 200, or 2000 drops | Drops = (10-FSR)*10 | Drops = (10-FSR)^10^/2e6 | Drops = 100/SRA^2^ | Drops = 1/SRA*10 | Drops = 100*2^((1-SRA)*(1-FSR)) |
| *Lysobacter* | 2 | 20 | 200 | 2000 | 2 | 2 | 46 | 2 | 2 | 3 | 200 |
| *Pseudomonas* | 2 | 20 | 200 | 2000 | 2 | 2 | 59 | 26 | 2 | 2 | 16 |
| *Sphingomonas* | 2 | 20 | 200 | 2000 | 2 | 2 | 71 | 168 | 2986 | 5464 | 27 |
| *Burkholderia* | 2 | 20 | 200 | 2000 | 2 | 2 | 74 | 243 | 2 | 2 | 34 |
| *Rhizobium* | 2 | 20 | 200 | 2000 | 2 | 2 | 85 | 1033 | 2 | 99 | 73 |
| *Bacillus* | 2 | 20 | 200 | 2000 | 2 | 200 | 96 | 3401 | 2 | 2 | 132 |
| *Chitinophaga* | 2 | 20 | 200 | 2000 | 2 | 200 | 98 | 4200 | 2 | 2 | 173 |
| *Mucilaginibacter* | 2 | 20 | 200 | 2000 | 2 | 200 | 99 | 4520 | 2 | 4 | 184 |
| *Bosea* | 2 | 20 | 200 | 2000 | 2 | 200 | 99 | 4730 | 2 | 65 | 192 |
| *Rhodococcus* | 2 | 20 | 200 | 2000 | 2 | 200 | 99 | 4731 | 21 | 457 | 192 |
| *Paenibacillus* | 2 | 20 | 200 | 2000 | 2 | 200 | 99 | 4743 | 2 | 2 | 187 |
| *Niastella* | 2 | 20 | 200 | 2000 | 2000 | 2000 | 100 | 4987 | 2 | 6 | 197 |
| *Variovorax* | 2 | 20 | 200 | 2000 | 2000 | 2000 | 100 | 4988 | 2 | 4 | 196 |
| *Arthrobacter* | 2 | 20 | 200 | 2000 | 2000 | 2000 | 100 | 4999 | 2 | 2 | 168 |
| *Bradyrhizobium* | 2 | 20 | 200 | 2000 | 2000 | 2000 | 100 | 5000 | 2 | 71 | 200 |
| *Methylo-*  *bacterium* | 2 | 20 | 200 | 2000 | 2000 | 2000 | 100 | 5000 | 2 | 71 | 200 |
| *Mycobacterium* | 2 | 20 | 200 | 2000 | 2000 | 2000 | 100 | 5000 | 70 | 837 | 200 |
| *Brevibacillus* | 2 | 20 | 200 | 2000 | 2000 | 2000 | 100 | 5000 | 5739 | 7575 | 200 |

^a^FSR = F/S ratio, as defined in Supplemental Table 1. The minimum number of drops per organism was set at 2.
