# Supplementary material for: A Reproducible and Tunable Synthetic Soil Microbial Community Provides New Insights into Microbial Ecology
Source: mSystems. 2022 Dec 6;7(6):e00951-22. doi: 10.1128/msystems.00951-22 (PMC9765266; doi:10.1128/msystems.00951-22)
Supplement: TEXT S1 [file msystems.00951-22-s0009.docx]

**A. Synthetic community generation protocol**

1. Start liquid cultures of the soil isolates in 14-mL culture tubes containing 3-5 mL 0.1X Reasoner’s 2A (R2A) (Teknova, cat # R0005) media 5-7 days prior to community generation. Isolate cultures are grown in aerobic conditions, 30 °C, without shaking.
   1. Cultures can also be streaked on R2A agar to check for contamination before starting liquid cultures.
2. Prepare an experiment plate map detailing the planned community compositions and locations within the plate. This will be useful later when assembling communities.
   1. Make sure to include negative control wells of sterile media.
3. On day of community generation, measure the OD_600_ of each isolate culture. After subtracting the value of sterile 0.1X R2A, dilute each isolate to OD_600_ 0.025.
4. Coat the lid of a 96-well plate with 3mL of an aqueous solution with 20% ethanol and 0.01% Triton X-100 (Sigma, cat # X100-100ML) for 30 sec. Then remove coating liquid and allow the lid to air-dry for 30 min under a UV light (to prevent bacterial contamination).
   1. This hydrophobic coating prevents condensation forming on the lid during community growth.
5. Add 200 µL/well of liquid 0.1X R2A to the wells of the plate. This is the target plate for the CellenONE machine.
6. Assemble the community by hand or through use of a SCIENION CellenONE machine (https://www.scienion.com/). See below for details.
   1. Take a Time 0 OD_600_ reading.
7. Grow the communities for desired length of time at in aerobic conditions, 30 °C, without shaking. To reduce evaporation from the edge of the plate, place the plate on top of 4 100mm-diameter Petri dishes (2 stacks of 2 dishes) filled with ~20 mL water each.
8. Monitor community growth with OD_600_ readings.
9. At the desired time point, harvest community samples. For metagenomics, communities can be frozen at -20 °C until they are processed for sequencing libraries.

**B. Community assembly with a CellenONE machine**

1. Fill the CellenONE wash bottle and empty the waste bottle, if necessary.
2. Turn on and prime the CellenONE machine according to manufacturer’s instructions. Use freshly filtered and degassed MilliQ water as system liquid.
3. Set up the machine with a medium-size CellenONE piezo dispense capillary (PDC) (Scienion, cat # P-20-CM).
4. Transfer diluted isolates into a 96- or 384-well probe plate, one isolate per well. Set the probe plate in the probe plate holder in the CellenONE machine.
   1. If using a 384-well plate, might need more than one well to have enough volume for the slow-growing isolates.
5. Place the target plate (prepared above) in the target plate holder.
6. Program the CellenONE machine to dispense the appropriate number of drops for each isolate.
   1. Steps for an individual isolate:
      1. Draw up volume for a single isolate from a well of the probe plate (up to 30 µL)
      2. Use drop camera to check for droplet formation and integrity. If droplet is not round and centered, adjust PDC parameters.
      3. Dispense the correct number of drops to each well of the target plate
      4. Use drop camera again to confirm droplet formation and integrity. If droplet has lost integrity, it is possible that not all target wells got the correct number of drops.
      5. Flush PDC with 0.5 mL system liquid
      6. Repeat for the same isolate with a fresh probe plate well if dispensing a large number of drops. Each drop ~400 pL, so 30 µL is enough for 75,000 drops.
      7. Repeat steps i-vi for each isolate
   2. After dispensing all isolates, dispense 200 drops of sterile media into each negative control well.
   3. The CellenONE software should come with a 96-well target and 96-well probe plate available as options. If not, work with a Scienion representative to set that up.
   4. Refer to the plate map created in step A2 to determine how many drops of each isolate go in each target plate well. The number of drops per isolate used for the communities in this study can be found in Supplemental Table 3.
   5. In our experience, the CellenONE machine can error when dispensing 2000 drops into many wells of a 96-well plate. If this occurs repeatedly, 2 µL of diluted isolate can be added to the wells with a multichannel pipette.
7. After all isolates have been dispensed into the target plate, remove the plate from the CellenONE machine and cover with the coated plate lid. Incubate and monitor as described above.

**C. Community assembly by hand**

1. Mix diluted isolates in correct proportions with micropipettes. Since pipetting by hand on the picoliter scale is impossible, scale volumes up as necessary. Make sure proportions between organisms are the same as in Supplemental Table 3.
   1. In this study, hand-assembled communities were generated with the following conversions:
      1. 2000 drops 🡪 600 µL
      2. 200 drops 🡪 60 µL
      3. 2 drops 🡪 0.6 µL
   2. Make one community mix per biological replicate.
2. Inoculate 1 µL per well in the 96-well plate prepared in step A5.
3. Cover plate with the coated plate lid. Incubate and monitor as described above.
